# Seizure as a Rare Initial Manifestation of Empty Sella Turcica Associated With Idiopathic Intracranial Hypertension: A Case Report

**DOI:** 10.1155/carm/4244868

**Published:** 2026-06-27

**Authors:** Karim Lakhdar, Zineb Moudafia, Mohamed Amine Elhasnaoui, Ounci Essaad, Rajae Alkouh, Siham Rachidi Alaoui, Smael Labib

**Affiliations:** ^1^ Intensive Care Unit B, Mohammed VI University Hospital Center, Abdelmalek Essaadi University, Tetouan, Morocco, uae.ma; ^2^ Radiology Department, Mohammed VI University Hospital Center, Abdelmalek Essaadi University, Tetouan, Morocco, uae.ma

## Abstract

Empty sella turcica (EST) is a radiological condition frequently associated with idiopathic intracranial hypertension (IIH), typically presenting with headache and visual disturbances. Seizures are rarely reported in this context. We report the case of a 41‐year‐old woman presenting with new‐onset generalized tonic–clonic seizures preceded by a 15‐day history of severe headache. Neurological examination was unremarkable, while fundoscopy revealed bilateral papilledema. Brain imaging demonstrated an empty sella, and lumbar puncture confirmed elevated intracranial pressure (32 cm H_2_O) with normal cerebrospinal fluid composition, consistent with IIH. Endocrine evaluation showed isolated thyrotropic insufficiency. The patient was treated with acetazolamide and topiramate, leading to rapid clinical improvement, normalization of intracranial pressure, and no recurrence of seizures during follow‐up. This case highlights that seizures may represent an unusual initial manifestation of IIH associated with EST and underscores the importance of considering intracranial hypertension in the diagnostic workup of unexplained seizures.

## 1. Introduction

Empty sella turcica (EST) is defined by the herniation of the subarachnoid space into the sella turcica, resulting in partial or complete filling with cerebrospinal fluid and compression of the pituitary gland [1]. It may be classified as primary, often associated with idiopathic intracranial hypertension (IIH), or secondary to pituitary pathology, surgery, or radiotherapy. With the increasing use of magnetic resonance imaging, EST is more frequently identified, often as an incidental finding [2–4].

tClinical manifestations of EST are heterogeneous and may include headache, visual disturbances related to papilledema, and endocrine abnormalities [3, 4]. IIH, commonly associated with EST, typically presents with chronic headache and visual symptoms, particularly in overweight women [2, 5]. However, seizures are not considered a classical manifestation of either EST or IIH and remain exceptionally reported in the literature.

Recent studies suggest that neurological manifestations of intracranial hypertension may extend beyond classical symptoms, although epileptic events remain rare and poorly understood [2, 5, 6]. The occurrence of seizures in this context raises questions regarding possible pathophysiological mechanisms, including cortical irritation and venous congestion [6].

Here, we report a case of generalized tonic–clonic seizures revealing EST in the setting of IIH, highlighting a rare and atypical clinical presentation.

## 2. Case Presentation

A 41‐year‐old woman with a history of well‐controlled arterial hypertension, treated with perindopril arginine (10 mg/day) and amlodipine (5 mg/day), with home blood pressure measurements consistently ranging between 120 and 135/70–80 mmHg, and no prior neurological history, was admitted following two generalized tonic–clonic seizures lasting approximately five minutes each, separated by a period of full recovery of consciousness. These episodes were preceded by a severe, persistent headache evolving over 15 days, described as resistant to usual analgesics.

On admission, the patient was conscious, afebrile, and hemodynamically stable. Blood pressure was 130/70 mmHg. Neurological examination revealed no focal deficits or meningeal signs. Body mass index was 30 kg/m^2^.

**FIGURE 1:**
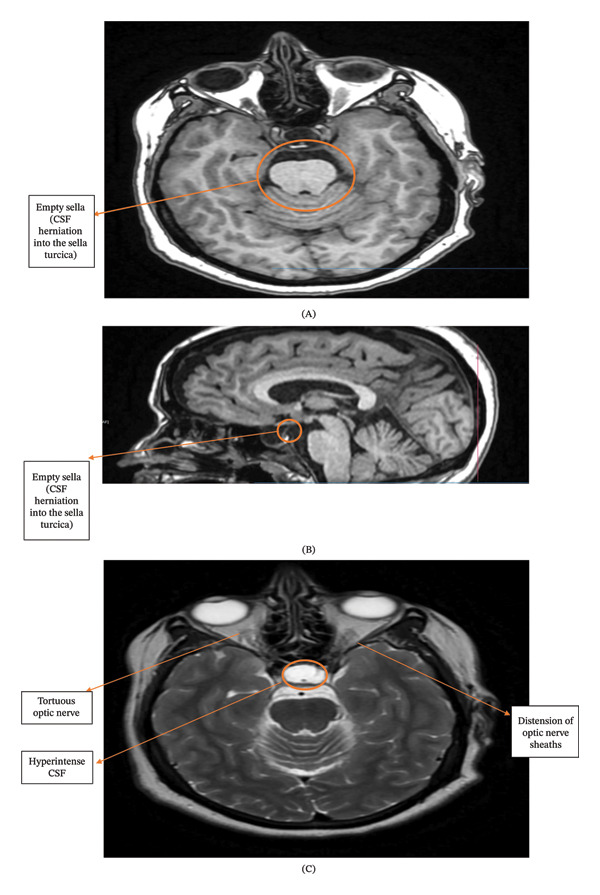
Neuroimaging findings of empty sella turcica and associated signs of idiopathic intracranial hypertension. (A) Axial T1‐weighted MRI showing cerebrospinal fluid filling the sella turcica with compression and flattening of the pituitary gland along the sellar floor. (B) Sagittal T1‐weighted MRI confirming sellar enlargement and cerebrospinal fluid herniation. (C) Axial T2‐weighted MRI demonstrating hyperintense cerebrospinal fluid signal within the sella, consistent with empty sella and bilateral distension of the optic nerve sheaths with tortuous optic nerve (consistent with idiopathic intracranial hypertension).

Fundoscopic examination showed bilateral Grade I papilledema. Brain computed tomography revealed an arachnoidocele with signs suggestive of intracranial hypertension. Magnetic resonance imaging confirmed EST and demonstrated additional signs suggestive of IIH, including bilateral optic nerve tortuosity and distension of the perioptic subarachnoid spaces (Figures 1A–C).

Electroencephalography was not performed because the patient remained neurologically intact after the event and neuroimaging revealed no structural epileptogenic lesion. Nevertheless, this represents a limitation of the present report.

Lumbar puncture demonstrated elevated intracranial pressure (32 cm H_2_O), with normal cerebrospinal fluid composition. Laboratory investigations were unremarkable except for isolated thyrotropic insufficiency, for which levothyroxine (75 μg/day) was initiated.

The patient was treated with acetazolamide (250 mg every 6 h) and topiramate (50 mg/day). Because the seizures were brief and self‐terminating, with complete recovery of consciousness between episodes, no emergency antiseizure treatment was required.

After 48 h, intracranial pressure decreased to 20 cm H_2_O, with marked improvement in headache intensity. No recurrence of seizures was observed during follow‐up, and no additional courses of acetazolamide were required.

Following treatment, the patient reported complete resolution of headaches, with no recurrence suggestive of uncontrolled systemic hypertension.

## 3. Discussion

EST is a common radiological finding frequently associated with IIH, particularly in overweight women. Its pathophysiology is thought to involve chronically elevated intracranial pressure leading to herniation of the subarachnoid space into the sella turcica and compression of the pituitary gland [1, 2, 7].

Secondary causes of EST, including prior cranial trauma, congenital syndromes such as septo‐optic dysplasia, and genetic conditions like KBG syndrome, were not supported by the patient’s history or clinical findings [1, 3, 4].

IIH typically presents with headache and visual disturbances related to papilledema, whereas seizures are not considered a classical manifestation of either EST or IIH and remain exceptionally reported in the literature. Nevertheless, rare cases of seizures associated with intracranial hypertension have been reported, suggesting a possible but poorly understood association [2, 5, 6]. Recent studies have highlighted that neurological manifestations of intracranial hypertension may extend beyond classical symptoms, although epileptic events remain exceptional [2, 5].

The diagnosis of IIH was supported by elevated opening pressure, papilledema, normal CSF composition, and absence of structural lesions on imaging.

Differential diagnoses of new‐onset seizures include primary epilepsy, metabolic disturbances, intracranial lesions, and vascular abnormalities [8]. In our case, these causes were excluded based on clinical evaluation, normal laboratory findings, and neuroimaging.

Several mechanisms may account for this presentation. Elevated intracranial pressure may lead to subtle cortical dysfunction through mechanical stretching of cortical structures, impairment of microvascular perfusion, and venous hypertension, thereby lowering the seizure threshold and facilitating neuronal hyperexcitability [2, 6, 7]. In addition, transverse sinus stenosis, frequently observed in IIH, may further contribute to altered cerebral hemodynamics and cortical excitability [6, 9, 10]. Nevertheless, the exact mechanism remains speculative because seizures are exceptionally rare despite the relatively high prevalence of IIH [2, 5, 6].

Although systemic arterial hypertension was present in our patient, no direct causal relationship between systemic hypertension and IIH has been established [2, 5]. The patient’s blood pressure was well controlled, making hypertension an unlikely contributor to intracranial pressure elevation.

However, establishing a causal relationship remains challenging, as EST is often incidental and seizures may occur independently [1, 3, 6]. In our patient, the absence of alternative etiologies and the favorable response to acetazolamide support a possible association, although causality cannot be definitively established. This case contributes to the limited body of evidence suggesting a potential, yet noncausal, association between IIH, EST, and seizure occurrence.

From a therapeutic perspective, acetazolamide remains the first‐line treatment for IIH [2, 5, 11] and was associated with rapid clinical improvement in our case. No long‐term antiseizure medication was initiated specifically for epilepsy because the seizure was considered an acute symptomatic event related to intracranial hypertension. Topiramate was prescribed primarily for IIH management and headache control, although its antiseizure properties were considered an additional benefit [12, 13].

The rapid improvement observed in our patient probably reflects early reduction of intracranial pressure and resolution of symptoms associated with the acute seizure episode rather than complete remission of IIH, which generally requires long‐term treatment and follow‐up.

Further studies are needed to better elucidate the underlying pathophysiological mechanisms linking intracranial hypertension and epileptic manifestations and to determine whether this association reflects a causal relationship or an incidental coexistence.

## 4. Conclusion

EST is a heterogeneous condition frequently associated with IIH. Although seizures are not a typical manifestation, their occurrence may represent a rare presentation. This case highlights the importance of thorough evaluation of intracranial pressure and sellar abnormalities in patients presenting with unexplained seizures.

## Author Contributions

Karim Lakhdar: conceptualization, supervision, and writing–original draft.

Zineb Moudafia: investigation, visualization, and data curation.

Mohamed Amine Elhasnaoui: data collection and clinical management.

Ounci Essaad: methodology and validation.

Rajae Alkouh: writing–review and editing.

Siham Rachidi Alaoui: imaging interpretation.

Smael Labib: supervision and final approval.

## Funding

No funding was received for this manuscript.

## Conflicts of Interest

The authors declare no conflicts of interest.
